# Phase noise matching in resonant metasurfaces for intrinsic sensing stability

**DOI:** 10.1364/OPTICA.510524

**Published:** 2024-03-08

**Authors:** Isabel Barth, Donato Conteduca, Pin Dong, Jasmine Wragg, Pankaj K. Sahoo, Guilherme S. Arruda, Emiliano R. Martins, Thomas F. Krauss

**Affiliations:** 1 School of Physics Engineering and Technology, University of York, Heslington, York YO10 5DD, UK; 2 Sao Carlos School of Engineering, Department of Electrical and Computer Engineering, University of Sao Paulo, Sao Carlos-SP 13566-590, Brazil

## Abstract

Interferometry offers a precise means of interrogating resonances in dielectric and plasmonic metasurfaces, surpassing spectrometer-imposed resolution limits. However, interferometry implementations often face complexity or instability issues due to heightened sensitivity. Here, we address the necessity for noise compensation and tolerance by harnessing the inherent capabilities of photonic resonances. Our proposed solution, termed “resonant phase noise matching,” employs optical referencing to align the phases of equally sensitive, orthogonal components of the same mode. This effectively mitigates drift and noise, facilitating the detection of subtle phase changes induced by a target analyte through spatially selective surface functionalization. Validation of this strategy using Fano resonances in a 2D photonic crystal slab showcases noteworthy phase stability ( $\sigma <10^{-4}\;\pi$ ). With demonstrated label-free detection of low-molecular-weight proteins at clinically relevant concentrations, resonant phase noise matching presents itself as a potentially valuable strategy for advancing scalable, high-performance sensing technology beyond traditional laboratory settings.

## 1. INTRODUCTION

Resonances in dielectric photonic crystals [1,2] and in plasmonic nanostructures [3] have been explored both theoretically [4,5] and experimentally for biochemical sensing [6,7]. The main advantage of these approaches [8] is the ability to sensitively detect specific molecules without the need for labeling while also allowing surface imaging and the detection of multiple biomarkers in parallel (multiplexing) [9,10]. Within these platforms, imaging-enabled multiplexed sensing is typically achieved via an intensity-based readout, either directly [9], via hyperspectral imaging [11] or, more recently, by spatially tuning the nanopatterns to translate spectral into spatial information [12–14]. Instead of these intensity-based readout techniques, phase-based readouts can provide superior performance [15]. Corresponding approaches have been demonstrated with gold nanohole arrays [16] and with dielectric gratings [17,18], targeted at high-performance, low-cost sensing technology (see Table S1 in Supplement 1 for comparison).

Interferometry relies on interfering a signal beam with a reference beam and determining their phase difference. One practical advantage of interferometry is the ability to use a camera for the readout, which obviates the need for a spectrometer, leading to the potential of low-cost, high-performance technology. However, interferometric techniques only advance the performance and applicability of resonant refractometric sensing if the readout stability is considered [19]. For example, while the incorporation of resonant sensors into a Mach–Zehnder interferometer [20] appears to be straightforward, the separation of the beam into different optical paths makes such a system prone to noise. In contrast, the well-known advantages of common-path interferometry approaches [16,17], where the signal- and reference-beams travel along the same path, provide higher stability [19]. The use of a resonance greatly enhances the phase-sensitivity of the sensor compared to the non-resonant case because of the associated large phase shift [21]. Conventionally, this phase shift is then referenced against a non-resonant background [20]. Interfering a resonant with a non-resonant signal, however, increases the system noise because both signals respond differently to external influences, such as temperature fluctuations or mechanical vibrations. Therefore, we use a previously developed common-path interferometric readout [21] as the means to implement the concept of “resonant phase noise matching” that we introduce here. The idea is to intrinsically reference noise by probing the phase difference between two equally sensitive, orthogonal components of the same resonance mode. We design a square lattice nanohole array, which results in mode degeneracy [22,23] when illuminated with unpolarized light. Since both resonance components in the metasurface respond identically to external influences, the noise contribution of these external influences is removed and does not contribute to the measured phase. The principle can be understood as conceptually similar to sensing with bi-modal waveguides [24–26] adapted to resonant metasurfaces.

**Fig. 1.**
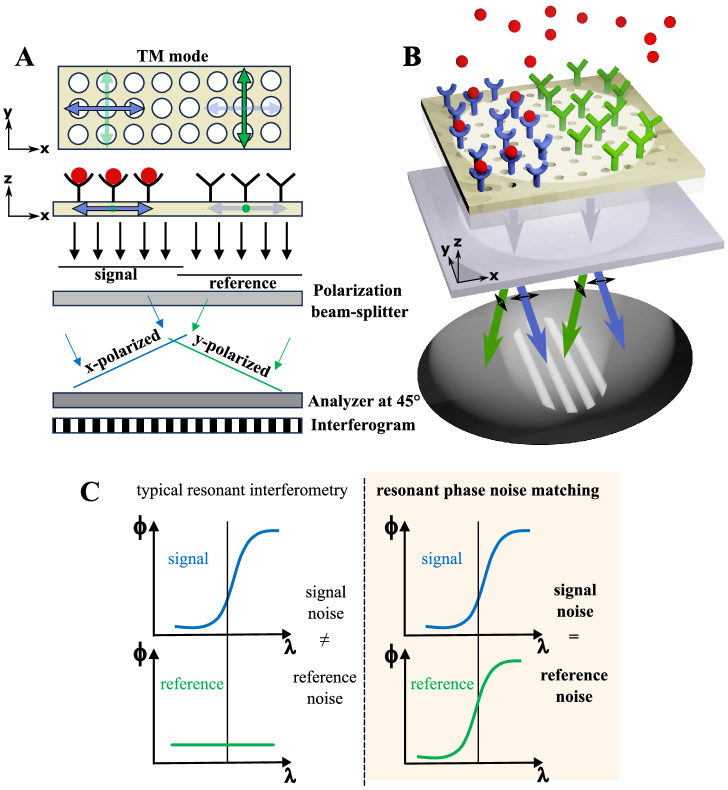
Simplified schematic illustration of resonant phase noise matching based on mode degeneracy. (A), (B), The sensor area is functionalized with specific antibodies (signal, blue) that capture the targeted biomarker (red), while the adjacent sensor area is non-specifically functionalized (reference, green). The phase difference ΔΦ is measured by splitting the orthogonal components of the resonantly reflected light, interfering them using an analyzer and capturing the resulting interferogram as a spatial fringe pattern. ΔΦ is only capturing specific biomarker binding, while non-specific binding and temperature drift does not contribute to ΔΦ since the mode is referenced with itself. The illumination path is not shown for clarity; see Supplement 1, SI 3 for the details of the optical setup. (C), Principle of resonant phase noise matching with an equal phase response to various sources of noise in the signal and reference, which leads to noise cancellation versus typical resonant interferometry with a “flat” reference (e.g., Michelson interferometry), where the noise in the resonantly reflected signal is different from the noise of the external reference.

Figures 1A and 1B illustrate this approach by visualizing the TM mode degeneracy ( x - and y -components), the sensor areas with specific (signal) and non-specific (reference) antibodies, and polarization beam-splitting, which is designed to spatially overlap the information originating from these adjacent surface areas. While both the x - and y -components of the TM mode are excited in both surface areas with a single beam, the polarization beam-splitting spatially overlaps only the x -component of one area with the y -component of the other area (middle blue and green arrows in Fig. 1B). The respective orthogonal components are present but do not overlap or contribute to the measured signal (outer blue and green arrows in Fig. 1B). The overall signal, extracted from lateral fringe movement in the interferogram, in principle registers exclusively the phase shift due to specific binding of the target markers (red in Fig. 1), while noise and drift are self-referenced. The measured phase shift ΔΦ is proportional to the effective index difference of the mode components $\Delta n_{eff}$ in the two sensing areas, where the sensitivity scales with the bandwidth Δλ of the resonant mode such that $\Delta \Phi =(\lambda /\Delta \lambda )\;\Delta n_{eff}$ . For an intuitive analogy of this principle of phase noise matching and why it intrinsically reduces noise compared to an approach that probes the phase difference between a resonance and a “flat” background or between two resonances with different sensitivities, please refer to the Supplement 1, SI 13.

## 2. RESULTS

### A. Photonic Crystal Resonance Characterization

**Fig. 2.**
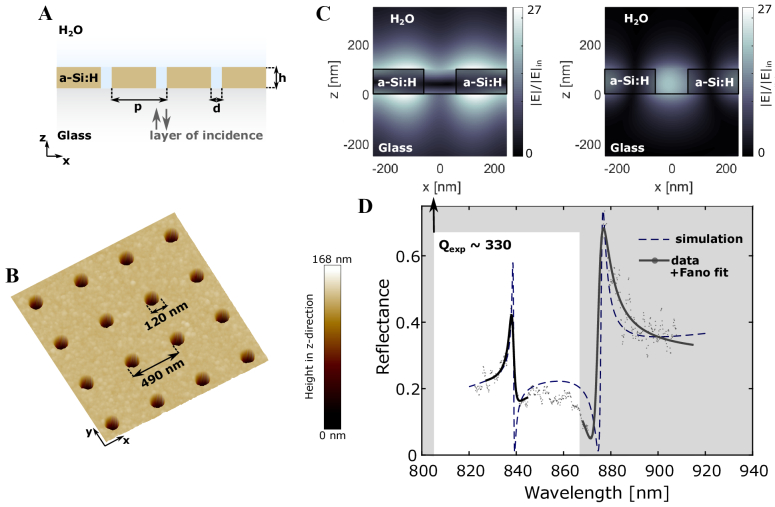
Photonic crystal slab design and characterization. Two-dimensional photonic crystal slab with a square lattice array realized in hydrogenated amorphous silicon (a-Si:H) of thickness h=100nm on a borofloat substrate with a periodicity of p=490nm and hole diameter of d=120nm . (A) Schematic side-view; (B) AFM top view; (C) simulated field confinement of the supported modes [TM-like (left)/TE-like (right)] normalized to the incident field $E_{in}$ (D) spectra of the two supported modes, both simulated and measured. The TM-like mode at ∼840nm with a Q -factor of 330 (white window) is used throughout this work.

A hydrogenated amorphous silicon (a-Si:H) photonic-crystal slab with a square array of nanoholes (Figs. 2A and 2B) supports two modes in the near-infrared wavelength range. The choice of a-Si:H is motivated by its high refractive index of n∼3.5 , which leads to stronger mode confinement, resulting in higher surface sensitivity (Supplement 1, SI 14) while exhibiting sufficiently low absorption loss [6] ( $k∼10^{-3}$ ) in the near-IR wavelength range to support high-quality factor resonances (Fig. 2D).

The Q -factor of the TM mode at the resonance wavelength $\lambda_{TM}^{peak}∼840\;nm$ of $Q_{TM}^{exp}∼330$ is higher than the Q -factor of the TE mode at $\lambda_{TE}^{peak}∼875\;nm$ with $Q_{TE}^{exp}∼166$ (Supplement 1, SI 8, Fano fitting and Q -factor extraction). A higher Q -factor corresponds to higher phase sensitivity ( $S_{\Phi }\propto S_{\lambda }/FWHM$ , see Supplement 1, SI 1) and the field confinement of the TM mode has preferable characteristics regarding the field overlap for surface sensing (Fig. 2C). Therefore, we design the nanohole array such that $\lambda_{TM}^{peak}$ corresponds to the peak wavelength of the LED source we use (Supplement 1, SI 6). Note that the Q -factor of the TM mode in comparable systems can reach higher experimental values [6] while maintaining a reasonable amplitude [27], especially in silicon nitride platforms ( Q∼1000 [17]). We chose not to work with such high Q -factors because we aim to match the resonance bandwidth with the bandwidth of the 1 nm bandpass filter implemented in combination with the LED. Using an LED removes the issues associated with highly coherent sources [28], such as speckle and background fringes, while further enabling wavelength tunability by rotation of the bandpass filter (Visualization 1). With these aims in mind, a moderate Q -factor of $Q_{TM}^{exp}∼330$ in combination with a bandpass filter of 1 nm bandwidth results in a sufficient resonant signal-to-background ratio (SBR) for high-contrast interferograms with a fringe visibility of ∼80% (Supplement 1, SI 11).

### B. Phase Response, Sensitivity, and Noise

The TM mode at the Γ -point is excited with unpolarized light to ensure the required degeneracy (Figs. 1 and 2A; Supplement 1, SI 3 for optical setup details). Note that the metasurface acts as a grating coupler such that the x and y far-field components are coupled to the TM mode that is predominantly oscillating in z in the near-field (Fig. 2C). The resonantly reflected light is directed towards the polarization beam-splitter (birefringent prism) where a small shear between orthogonally polarized x - and y -components is created such that the resulting diverging beams partially overlap in the image plane. An analyzer at 45° is placed in front of the camera to allow the orthogonally polarized components to interfere and create a spatial fringe pattern (Fig. 1). A phase shift between the sensitivity-matched mode-components in adjacent sensor areas then leads to a lateral shift of this fringe pattern [29].

**Fig. 3.**
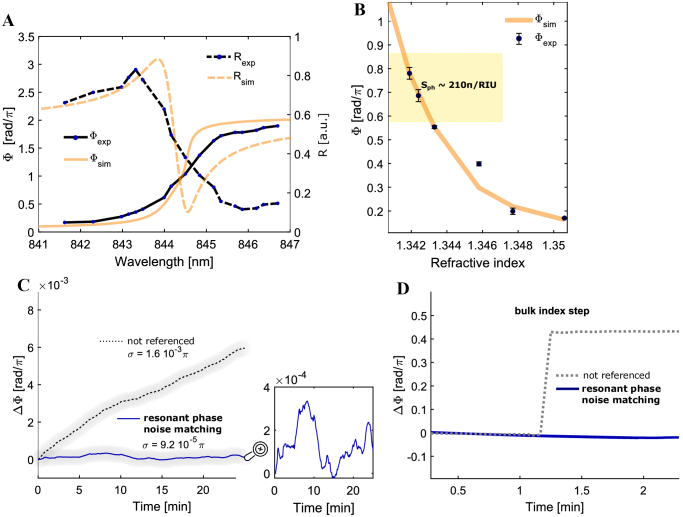
Experimental phase response, bulk sensitivity, and noise characterization. (A), Phase and reflectance as a function of wavelength. The wavelength is tuned by rotating the bandpass filter, and the reflectance and phase values are extracted from recorded interferograms. The resonance phase shift is here referenced to the shift observed off-resonance (reflection from un-patterned surface areas). See also Visualization 1. (B), Phase in response to bulk refractive index changes. The index was changed by flowing glucose solutions of different concentrations over the sensor surface and detecting the response over time (Supplement 1, SI 7). The range shown here corresponds to the relevant range around the peak of the resonance. The phase values are the mean obtained from the phase over time at constant index and the error bars represent the standard deviation. (C), Phase over time while the refractive index is not deliberately altered ( $H_{2}O$ index of 1.333) to characterize the system noise with and without self-referencing. This graph shows the difference between the “sharp-flat” and the “sharp-sharp” approaches. The apparent drift is clearly removed, and the nature of the remaining “sharp-sharp”-noise is shown in the zoom inset (see also Supplement 1, SI 16). (D), Phase in response to bulk index change with and without self-referencing to show that self-referencing removes bulk effects, which would otherwise disturb the specific surface sensing.

Figure 3 shows the extracted phase information in response to sweeping the wavelength of the illumination beam (Supplement 1, SI 3) and to bulk refractive index changes. Because the self-referenced interferometry approach is based on referencing two orthogonal components of the same mode with the same sensitivity (Fig. 1C), wavelength changes and bulk index changes do not introduce shifts in the detected interferogram (Fig. 3D). It is therefore not possible to characterize the phase response of the mode with this approach. However, the FOV consists of areas where one mode overlaps with the reflection from the un-patterned slab in addition to the overlap with itself. Therefore, by adjusting the analyzer such that the resonantly reflected light intensity matches the background intensity to achieve sufficient contrast (Supplement 1, SI 11), phase shifts in response to wavelength sweeping can be measured via mode-background interference, purely for the purpose of the mode phase response characterization, not for the biosensing itself.

The measured phase curve (Fig. 3A) deviates from the π phase jump of a single driven oscillator with Lorentzian line shape [30,31]. Resonances in 2D photonic crystal slabs such as the nanohole array used here result from the interaction of two resonances, i.e., (a) the guided-mode resonance produced by the periodic structure and (b) the Fabry–Perot resonance of the slab, which combine to form a well-known Fano resonance. While the underlying mechanism of a Fano resonance is well understood [30,31], the corresponding phase behavior is rarely shown and discussed more specifically for sensing. Due to the nature of Fano resonances, the phase response is not symmetric around the reflectance peak maximum since the interference of two resonances changes the overall phase response [30,32]. Here, it is the interference of the Fabry–Perot resonance in the slab with the guided-mode resonance that leads to the observed deviation from a symmetric π phase jump (see Supplement 1, SI 2 for detailed explanation).

The experimental bulk phase sensitivity in the steepest range of the phase curve is ∼210π/RIU in accordance with the response predicted by rigorous coupled wave analysis (RCWA; see Section 3 and Fig. 3B). We choose this range for the sensing experiments by finding the linear range of the phase response corresponding to the intermediate intensity value between the minimum and maximum reflectance of the resonance (Fig. 3A).

Figure 3C shows how the self-referencing based on mode degeneracy leads to a stable baseline, by intrinsically accounting for drift. The theoretically predicted result of the self-interference would be a perfectly flat response. While the inset of Fig. 3C shows a low degree of remaining noise, the resulting standard deviation is $\sigma =9.2\cdot 10^{-5}\pi$ , which is lower than any previously reported value in comparable platforms (Supplement 1, SI 12), to the best of our knowledge. Importantly, this baseline is obtained in a system that is not temperature-stabilized and therefore drifting over time. The drift is characterized (Fig. 3C, dashed line) by extracting the unreferenced baseline from the interferogram that is generated due to the interference of the resonant signal with light reflected from the un-patterned slab rather than itself. In addition, Figure 3D shows the response to a deliberate, sudden bulk refractive index change to highlight the cancelling of unwanted phase changes by the self-interference. While the bulk index change was here induced by changing the liquid on the sensor surface, such bulk index changes are likely to occur during a sensing experiment due to temperature drift and the accompanying changes in density registered as phase drift over time, such as that seen in Fig. 3C.

The resulting overall bulk refractive index limit-of-detection is $LOD=3\sigma /S_{ph}=1.2\cdot 10^{-6}$ RIU, which represents an improvement when compared to similarly simple systems with out-of-plane coupling (Supplement 1, SI 12).

When illuminating the nanohole array, the optical alignment must ensure that the degeneracy is maintained. Otherwise, when the degeneracy is lifted due to angular deviation ( $k_{x}\;\neq \;k_{y}$ ), different points on the phase curves are probed for the signal and reference, leading to potentially different phase sensitivities. Typical alignment procedures are sufficient to achieve this, yet we have found it helpful to implement a spatially tuned nanohole array approach [6] on the same chip to visualize and optimize the degeneracy during alignment (Supplement 1, SI 15). Increasing the angular tolerance of the resonant mode might also be possible if required for future work, e.g., by using flatband resonances [33], a perturbation approach [34], or anapole modes.

### C. Protein Sensing

**Fig. 4.**
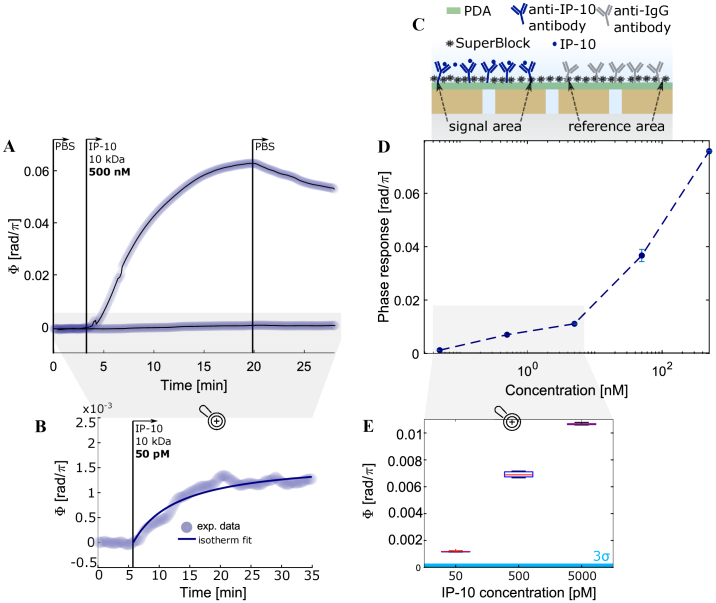
Proof-of-principle sensing of the small protein IP-10 at various concentrations. The sensor area was functionalized with antibodies specific to IP-10 while the reference sensor area was functionalized with a non-specific reference-IgG. (A), Phase response to the highest (500 nM) concentration and, (B), enlarged section of the response to the lowest tested IP-10 concentration (50 pM). (C), Schematic of the sensor surface after functionalization and biomarker binding. (D), Phase in response to various IP-10 concentrations plotted on a semi-logarithmic scale. An equilibrium constant $K_{D}=60\;nM$ is estimated from a Langmuir fit [42] of the same data in Supplement 1, SI 5). N=1 , error bars show confidence of signal extraction and do not correspond to repeat experiments. (E), Boxplot of the phase over time at saturation in response to the three lowest concentrations and indication of the 3σ noise level in comparison, showing that the 50 pM response is well above the LOD.

The low-noise baseline should translate into the ability to detect the binding of biomarkers with low molecular weight at low concentrations. To test this, we measured the phase response when flowing various concentrations of the clinically relevant protein called interferon γ-induced protein 10 (IP-10/CXCL10) over the functionalized [35] sensor surface. The alteration of CXCL10 levels is generally linked with infectious disease [36,37] and is currently emerging as a potential biomarker for several diseases [38–40], but a label-free detection of such a small protein with a molecular weight of 10 kDa is challenging. In Fig. 4, we show that a concentration of 50 pM results in a phase shift of $∼13\cdot 10^{-4}\;rad/\pi$ within ∼15min . This value is almost 5× above the 3σ noise level of $2.8\cdot 10^{-4}\;rad/\pi$ , suggesting an LOD in the range of a few tens of pM, which is sufficiently low to distinguish normal clinical levels of CXCL10/IP-10 ( ∼600pg/mL corresponding to ∼60pM ) from elevated levels (e.g., ∼3000pg/mL corresponding to ∼300pM ) [41]. This is the first time to our knowledge that CXCL10/IP-10 is shown to be detectable at clinically relevant concentrations using a label-free approach.

The surface functionalization for the experiments shown in Fig. 4 was performed with a microfluidic approach to introduce the specific and non-specific antibodies to the adjacent areas with a pre-treatment based on polydopamine-coating [35]. We have also tested the “spotting” of antibodies using precision dispensing of ultra-low antibody solution volumes for the same purpose (see Supplement 1, SI 9), and both approaches are applicable.

The issue of an inherently limited resonance dynamic range ( $\Delta n_{\Phi }^{dyn}\propto FWHM$ ; Supplement 1, SI 1 and Eq. S2) can be either compensated by wavelength tunability using a broadband source in combination with a bandpass filter or the fabrication of detuned nanohole arrays within the FOV to probe consecutive ranges of the phase curve [17]. However, we note that an extension of the dynamic range is not required when aiming to detect proteins, especially at low concentrations. A saturation of the surface with the large immunoglobulin G (IgG, 150 kDa) results in a phase shift of less than 0.4π (SI 9) while maintaining measurable resonance amplitudes, meaning a typical phase shift lies within the range of a single resonance width without extending the dynamic range.

### D. Discussion

The main novelty of the work presented here is the idea to *optically* reference two components of the same resonant mode and thereby, due to their equal sensitivity, remove system drift and noise without signal subtraction and typical referencing. We achieve this by implementing a 2D photonic crystal slab with its degenerate eigenstates into a polarization-sensitive, common-path interferometric readout setup. While we previously demonstrated resonant common-path interferometry with high- Q resonances in dielectric gratings [17], “resonant phase noise matching” is here introduced and leads to the lowest phase noise ( $\sigma <10^{-4}\pi$ ) in any system of comparable complexity that we could find (i.e., phase-sensitive plasmonic nanohole arrays and interferometric waveguide sensing; see Supplement 1 , SI 12 and Table S1). While high Q -factor resonances and laser diodes were required in previous work [17], it is the resonant phase noise matching that here leads to a comparable or better performance ( $LOD∼10^{-6}$ ) with moderate Q -factor resonances and incoherent light sources. The approach we now describe is therefore more generally applicable and could lead to a wider use of resonant interferometry, both for plasmonic and dielectric sensing, since it combines state-of-the-art performance with greater tolerance in terms of nanofabrication, optical alignment, and light-source requirements.

The only conceptual requirement for phase noise matching and intrinsic stability regarding the photonic platform/substrate is the presence of two orthogonal modes with equal phase sensitivity, generally provided by any square-lattice array with unpolarized illumination. This includes both dielectric and plasmonic square-lattice periodic arrays. While it is in principle beneficial for phase-sensitive platforms to employ high- to moderate- Q resonances, corresponding to high phase sensitivity, it is not a requirement for the applicability of our intrinsic, interferometric referencing methodology. Therefore, plasmonic platforms, typically providing lower Q -factors than dielectric platforms, could benefit from our approach as well. Moreover, the intrinsic noise-reduction could in part compensate the shortcomings of low- Q platforms and low-cost components. As an alternative to the here-described square-lattice array approach to achieve degeneracy, 1D gratings could also achieve de facto degeneracy by 90°-rotation of the grating vector in the signal- and reference-sensor areas.

While the phase sensitivity could be further increased by designing resonant surfaces supporting higher Q -factor resonances [43], considerations regarding the trade-off between resonance amplitude and Q -factor led to our design of a platform with moderate Q -factor providing relatively high reflectance values at the resonance wavelength. This deliberately lower Q -factor with the benefit of higher tolerance and amplitude of the here-chosen resonances compared to, e.g., our previous work [17] explains why the overall sensing performance is just better, rather than significantly better, despite the lower phase noise. Especially for low-cost platforms, sufficiently high resonance signals are essential to generate high contrast interferograms and allow the use of low-cost cameras. The intrinsic stability provided by resonant phase noise matching in combination with the ability to use standard CMOS chips can pave the way for an integration in low-cost sensing devices.

The remaining resonant noise (Fig. 3C) shows a frequency dependent behavior; similar to pink noise (Supplement 1, SI 16), which indicates that the remaining noise is limited by the camera [44,45].

The often-made assumption is that highly coherent light sources are required for interferometry results in laser-based implementation approaches with unwanted speckle and background noise generated by coherent reflections from optical components and interfaces. In contrast, using sources with the minimally required degree of temporal coherence benefits interferometric approaches by avoiding speckle-related noise, which has been widely accepted within the holographic imaging community [28]. This knowledge is not readily transferrable to resonant interferometry since the ideal light source bandwidth depends on the linewidth of the photonic resonance. A narrow resonance peak ideally is excited with a similarly narrow excitation bandwidth because non-resonant light adds to the background and reduces the interferogram contrast. Therefore, a broadband source ideally suited for non-resonant interferometry needs to be spectrally filtered for highly sensitive resonant interferometry. Here, we show that moderate Q -factor resonances can be interferometrically probed using a spectrally filtered LED.

One way of further miniaturizing and scaling the optical setup would be to replace the birefringent prism with a flat-optics metasurface for polarization-based beam-splitting [46,47]. Although the efficiency of flat-optics components is not yet expected to reach those of bulk optical components, the use of highly scalable silicon-based flat-optics components [48] for resonant interferometry are an exciting prospect for future non-laboratory sensing technology.

## 3. MATERIALS AND METHODS

### A. Sensor Chip Fabrication

We use commercial wafers consisting of a 100 nm thin film of hydrogenated amorphous silicon (a-Si:H) on a 500 µm glass substrate (Inseto, UK) and dice them into $(15\times 15)\;{mm}^{2}$ pieces before cleaning them by sonication in Acetone (ACE) for 10 min, rinsing in isopropanol (IPA), and placing them for 5 min in a plasma asher. The resist we use for electron-beam lithography (EBL) is AR-P 6200.13 from Allresist GmbH spun at 5000 rpm for 60 s and baked on a hotplate at 180° for 5 min. For charge dissipation during the EBL exposure, we use the conductive polymer AR-PC 5090 (Allresist GmbH) spun at 2000 rpm and baked on a hotplate at 90° for 2 min. The pattern is written with a Voyager EBL system (Raith GmbH, 50 kV) with a beam current of 135 pA and a dose of $215\;\text{µ}{C/cm}^{2}$ . Each sensor area consists of $(500\times 500)\;{\text{µ}m}^{2}$ arrays. After removing the charge dissipation layer in deionized water at room temperature for 2 min, we develop the pattern in Xylene for 2.5 min at room temperature and stop the development with a rinse in IPA. Next, the pattern is transferred into the a-Si:H layer by plasma-based reactive ion etching (RIE) using a gas mixture of ${CHF}_{3}$ and ${SF}_{6}$ at a ratio of 14.5 sccm:12.5 sccm. After etching for 1 min and 5 s with a DC bias of 188 V and a chamber pressure of 0.4 mbar, we remove the remaining resist with gentle sonication in 1165 Microposit Remover (Shipley) for 10 min at 50°C. The referenced sensor areas are chosen to correspond each to a write field, respectively, such that the alignment and therefore the dose are as similar as possible leading to the lowest possible difference in phase sensitivity. Differences in the dose would be detectable in the measured hole radius.

### B. Surface Chemistry

The sensor surface is functionalized by first creating a thin layer of polydopamine directly on the cleaned sensor surface. The cleaning is done either by Piranha or 30 min UV ozone and a series of washes (Hellmanex, MQ water) in gentle sonication to avoid damaging the sensor. The polydopamine (PDA) solution is then prepared by mixing 20 mg of dopamine hydrochloride (H8502, Sigma Aldrich) in 10 mL of Tris buffer at pH 8.5. After vortexing this solution, the whole sensor is immersed for 15 min and kept on a thermo-shaker for the duration of the incubation at room temperature. The shaking is chosen because it helps to supply the oxygen that is necessary for the reaction and because otherwise the precipitation during the reaction could lead to accumulation of unwanted particles on the sensor surface. The color of the solution turns slightly brown within 15 min if the reaction is successful. The sensor is then rinsed in Tris buffer twice and once in DI water before drying with nitrogen. This protocol is based on the previously published “Mussel-Inspired Surface Chemistry for Multifunctional Coatings” [49] and is almost identical to the protocol described in “Bio-inspired polydopamine layer as a versatile functionalization protocol for silicon-based photonic biosensors” [35].

### C. Optical Setup and Data Acquisition

See Supplement 1, SI 3 for details about the optical setup and data acquisition.

### D. Rigorous Coupled Wave Analysis Simulations

The simulations shown throughout this work were conducted using the open-source Stanford Stratified Structure Solver [50] ( $S^{4}$ ) implementation of RCWA. The corresponding Lua scripts to extract the spectra, phase response, sensitivity, and field confinements were written by the authors of this work and are available upon request.

### E. Optical Setup and Data Acquisition

The images containing the interferogram information are processed with a custom MATLAB implementation of FFT-based phase extraction. First, the relevant sensor areas are cropped, rescaled, FFT-filtered to generate clean interferograms without intensity inhomogeneities, and averaged along the axis perpendicular to the fringe periodicity. The extracted phase is then plotted after applying a Savitzky-Golday filter, which is based on convolution without information distortion. Note that phase unwrapping is not required here, in contrast to, e.g., waveguide-based interferometry approaches [51], since the detected phase shifts are restrained to the maximal phase shift of a single resonance ( <2π ).

## Data Availability

All data needed to evaluate the conclusions in the paper are present in the paper and/or the supplementary materials. Any additional data are available from the authors upon reasonable request. References [52–57] are cited in Supplement 1.

## Supplemental document

See Supplement 1 for supporting content.
