# Supplementary material for: Phase noise matching in resonant metasurfaces for intrinsic sensing stability
Source: Optica. 2024 Mar 8;11(3):354–61. doi: 10.1364/OPTICA.510524 (PMC11023067; doi:10.1364/OPTICA.510524)
Supplement: Supplementary file 1 [file optica-11-3-354-s001.pdf]

Supplemental Document

OPTICA

# Phase noise matching in resonant metasurfaces for intrinsic sensing stability: supplement

**Isabel Barth,[1,*] Donato Conteduca,[1] Pin Dong,[1] Jasmine Wragg,[1] Pankaj K. Sahoo,[1] Guilherme S. Arruda,[2] Emiliano R. Martins,[2] and Thomas F. Krauss[1]**

[1] *School of Physics Engineering and Technology, University of York, Heslington, York YO10 5DD, UK*
[2] *Sao Carlos School of Engineering, Department of Electrical and Computer Engineering, University of Sao Paulo, Sao Carlos-SP 13566-590, Brazil*
[*] *ibarth@mgh.harvard.edu*

This supplement published with Optica Publishing Group on 8 March 2024 by The Authors under the terms of the Creative Commons Attribution 4.0 License in the format provided by the authors and unedited. Further distribution of this work must maintain attribution to the author(s) and the published article's title, journal citation, and DOI.

Supplement DOI: https://doi.org/10.6084/m9.figshare.25041869

Parent Article DOI: https://doi.org/10.1364/OPTICA.510524

Supplementary Materials for

**Phase noise matching in resonant metasurfaces for intrinsic sensing stability**

Isabel Barth[*] *et al.*

*Corresponding author. Email: ibarth@mgh.harvard.edu

**This PDF file includes:**

Supplementary Text
Figs. S1 to S16

**Other Supplementary Materials for this manuscript include the following:**
Movies S1

**SI 1. Phase-sensitivity estimation based on spectral resonance information**

In the process of designing a resonant interferometry platform, it is possible to estimate the expected phase sensitivity of a resonance without having to simulate or measure the phase response to refractive index changes if the spectral sensitivity is known. The goal is to show the approximate relationship between the typically well-known spectral information and phase information to facilitate the design of phase-based sensing platforms. A simulated or experimentally obtained spectrum of the resonance peak allows to determine the FWHM. It is possible to estimate the phase sensitivity from the FWHM and spectral sensitivity of a resonance:

$$S_{\Phi}\,[\pi/RIU] \approx \frac{0.6\,\cdot\,S_{\lambda}\,[nm/RIU]}{FWHM\,[nm]} \qquad \text{Eq. S1}$$

The phase dynamic range (approximately linear range) of a single resonance can be defined as the index change Δn that results in a wavelength shift of Δλ = 1/2 FWHM:

$$\Delta n_{\Phi}^{dyn} \approx \frac{FWHM}{2\cdot S_{\lambda}} \qquad \text{Eq. S2}$$

These equations relate to the approximately linear range of a Lorentz resonance (Fig. S1) and together also illustrate the trade-off between phase sensitivity and dynamic range.

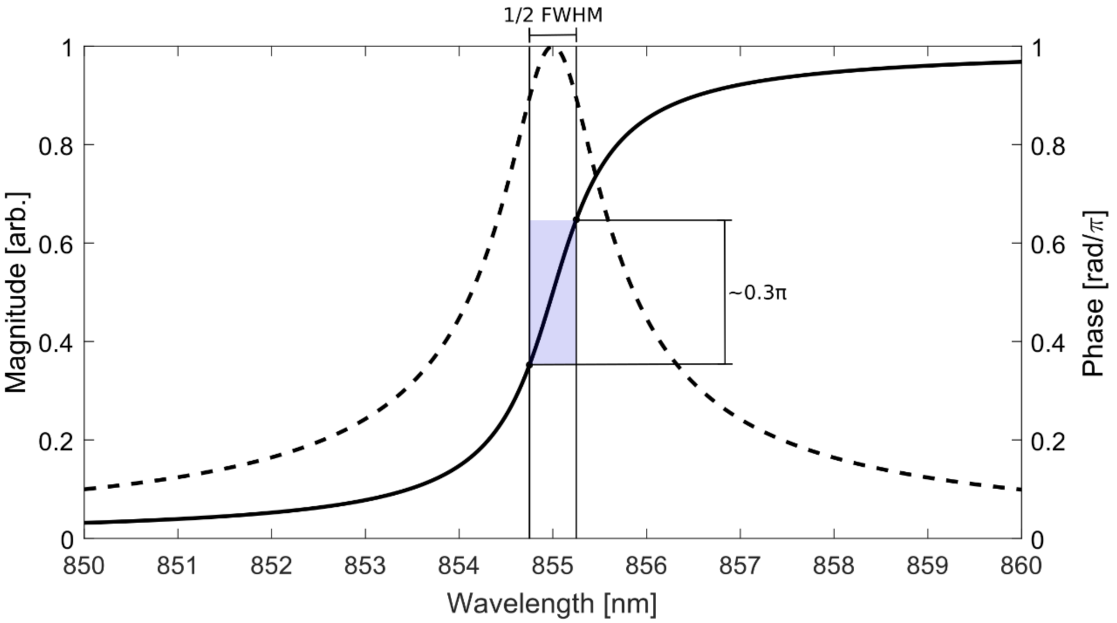


**Fig. S1. Phase and magnitude of Lorentz oscillator model.** For the illustration of the approximately linear range of the phase response within a range of FWHM/2.

**SI 2. Phase response of Fano resonances - explanation**

According to temporal coupled mode theory, the reflection channel is given by:

$$A(\omega) = r + f(\omega)(r \pm t)$$

The Fabry-Perot reflection coefficient $r$ is given by:

$$r = \frac{(1 - e^{-i\delta})\sqrt{R}}{1 - Re^{-i\delta}}$$

Where $\delta$ is the phase due to propagation inside the Fabry-Perot slab, and $R$ is the Fresnel coefficient between the slab and the external medium.

The Fabry-Perot transmission coefficient $t$ is given by:

$$t = \frac{(1 - R)e^{-i\frac{\delta}{2}}}{1 - Re^{-i\delta}}$$

Finally, the Lorentzian channel $f(\omega)$, which represents the GMR, is given by:

$$f(\omega) = -\frac{{Q_r}^{-1}}{2i\left(\frac{\omega - \omega_0}{\omega_0}\right) + {Q_r}^{-1}}$$

Notice that, for $\omega \ll \omega_0$, $f(\omega) \approx -\frac{{Q_r}^{-1}}{2i\left(\frac{\omega}{\omega_0}\right)}$, which is a phasor with phase equal to $\pi/2$, whereas for $\omega \gg \omega_0$, $f(\omega) \approx \frac{{Q_r}^{-1}}{2i\left(\frac{\omega}{\omega_0}\right)}$, which is a phasor with phase equal to $-\pi/2$. Therefore, $f(\omega)$ sweeps a phase shift of $\pi$ across the resonance.

The Fano resonance results from interference between the channel $r$ and the channel $f(\omega)(r \pm t)$. Since the only frequency dependent term is the Lorentzian term $f(\omega)$, and we have seen that $f(\omega)$ sweeps a $\pi$ phase shift, we should expect that $A(\omega)$ will also sweep a $\pi$ phase shift, since all other terms are constant. That is not the case, however, because apart from the phase sweep, the Lorentzian channel also changes amplitude, which affects the phase of $A(\omega)$.

We can identify two paradigmatic examples of phase sweep setting $R = 0.2$ and $\delta = \pi/2$. The figures below show the phase sweep associated with the $\pm$ sign in the equation for $A(\omega)$. The sign depends on the symmetry of the modes, and TE modes give positive sign, and TM modes give negative sign. For reference, the Lorentzian phase is also shown (the Lorentzian is obtained by setting $\delta = 2\pi$, which results in $r = 0$).

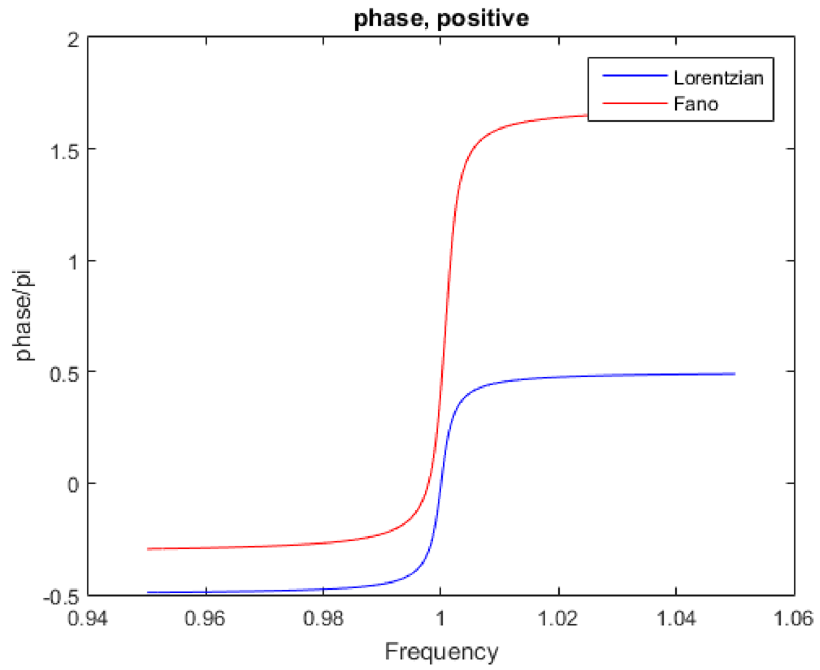


**Fig. S2**. **'positive' phase response of Lorentzian.**

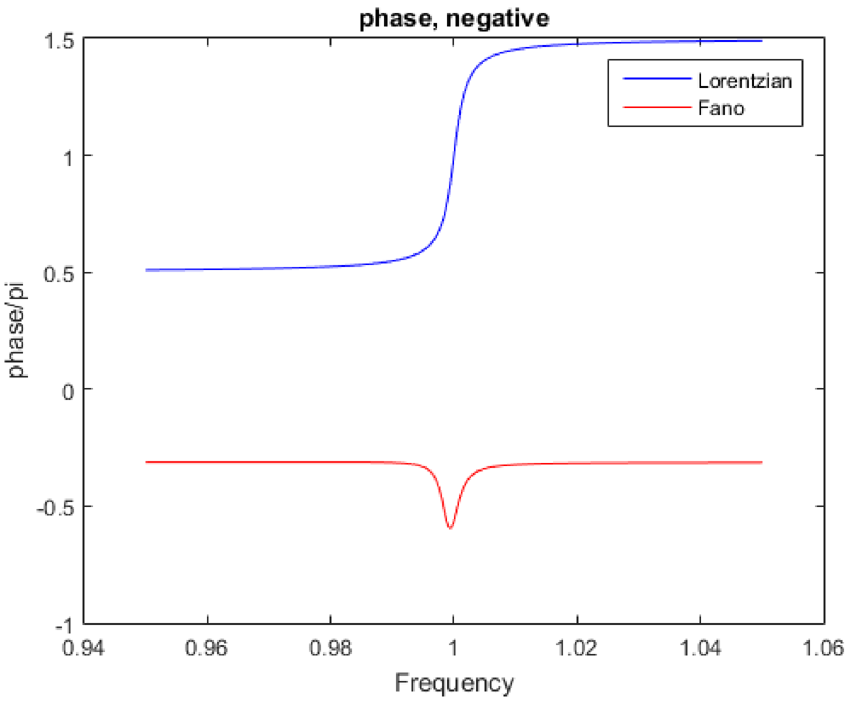


**Fig. S3**. **'negative' phase response of Lorentzian**

As can be seen from the Figures S2 a and b, there are two distinct phase behaviors. In the case when the "plus" sign is used, the Fano resonance makes a $2\pi$ phase sweep, whereas when the negative sign is used,

the Fano resonance sweep is only modest (these behaviors are not inherent to the plus or minus signs and may be inverted depending on the $\delta$ values used).
We can understand these behaviors by inspecting the interference between the channels.
First, focus attention on the green plots (positive) sign of Figure S4. The inset shows the corresponding phasors at different frequencies. The orange phasor is the reflection (Fabry-Perot) channel. Notice that, in this case, the Lorentzian channel (the green phasors) grows as it tends towards a p phase shift with respect to the reflection channel (the orange phasor). For example, at point 5 the phase of the Lorentzian (green) phasor is almost at a p angle with the FP (orange) phasor. Since the magnitude of the Lorentzian (green) phasor is large at this point, the phase of the Fano (blue) phasor is almost p shifted with respect to the FP. Since away from the resonance, the Lorentzian contribution is negligible, it follows that the Fano (blue) phase must eventually go back to the FP (orange) phase. Thus, it completes a 2p phase shift across the resonance.
This behavior pushes the phase of the total channel (the blue phasors) towards a full 2p phase loop across the resonance.

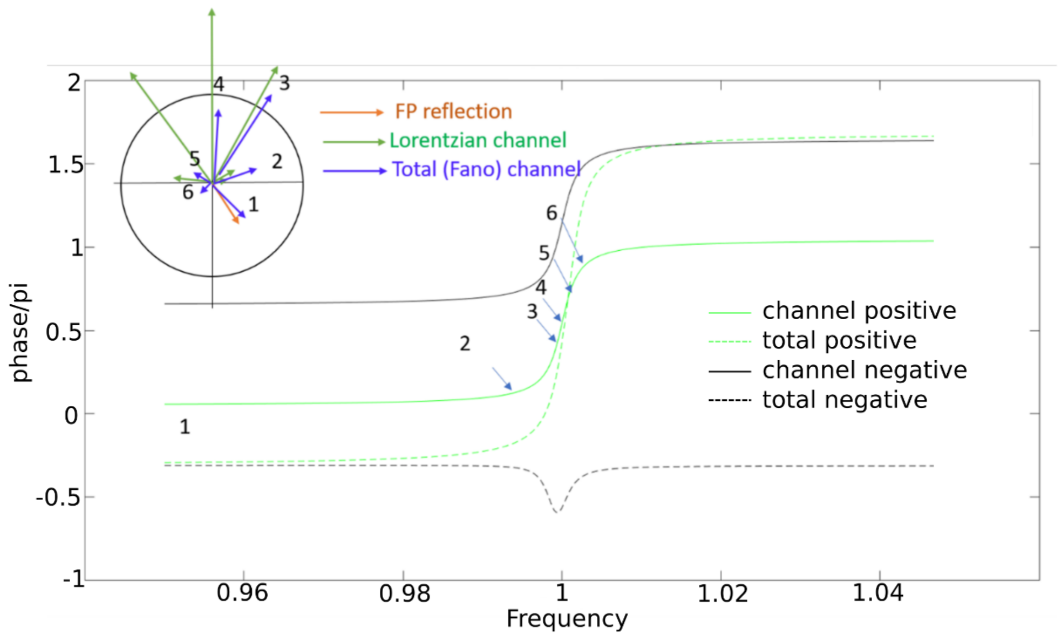


**Fig. S4**. **Loretz versus Fano phase**. Phase of the Lorentzian (GMR) channel in solid lines, and the total (Fano) phase in dashed lines. The green lines refer to the positive sign, and the black lines refer to the negative sign. Inset refers to the green curves.

Now contrast this behavior with the negative sign condition (black curves in Fig. S5).

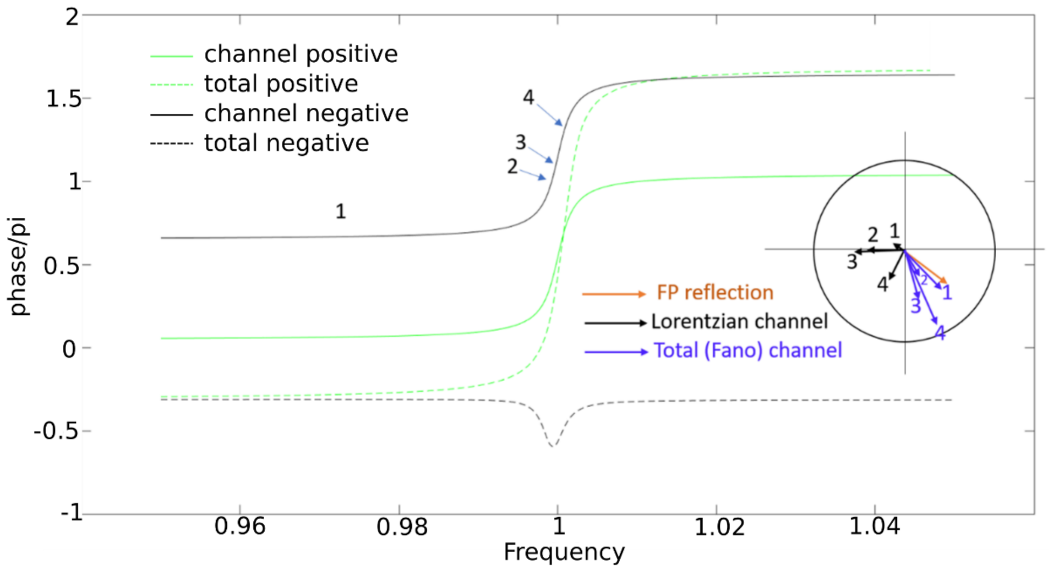


**Fig. S5**. **Loretz versus Fano phase**. Phase of the Lorentzian (GMR) channel in solid lines, and the total (Fano) phase in dashed lines. The green lines refer to the positive sign, and the black lines refer to the negative sign. Inset refers to the black curves.

Now there are two different features. First, the phase already starts at pi phase shift (see point 1), and then evolves TOWARDS the FP channel. As we move closer to resonance (around point 3), the contribution of the Lorentzian (black) channel increases, which pushes the Fano phase (blue) towards it. But since the Lorentzian channel now is moving towards the FP channel, the Fano phase (blue) quickly reduces, as the contribution of the Lorentzian channel diminishes. Notice that the phase at point 4 is already closer to the FP phase than the phase at point 3. That explains the “blip” in the phase of the negative condition (dashed black line).

The derivations above are meant to explain the phase response shown in Figure 3 of the main manuscript (Fig. S2) and going beyond this, highlight that different trends of the Fano phase response (i.e., Fig. S3) are also possible.

Figure S6 illustrates how resonances supported by the specific structure used in this work (Fig. 2 in main manuscript) with resonance wavelengths at different points of the FP continuum show interesting phase behaviors based on the above described interference effects.

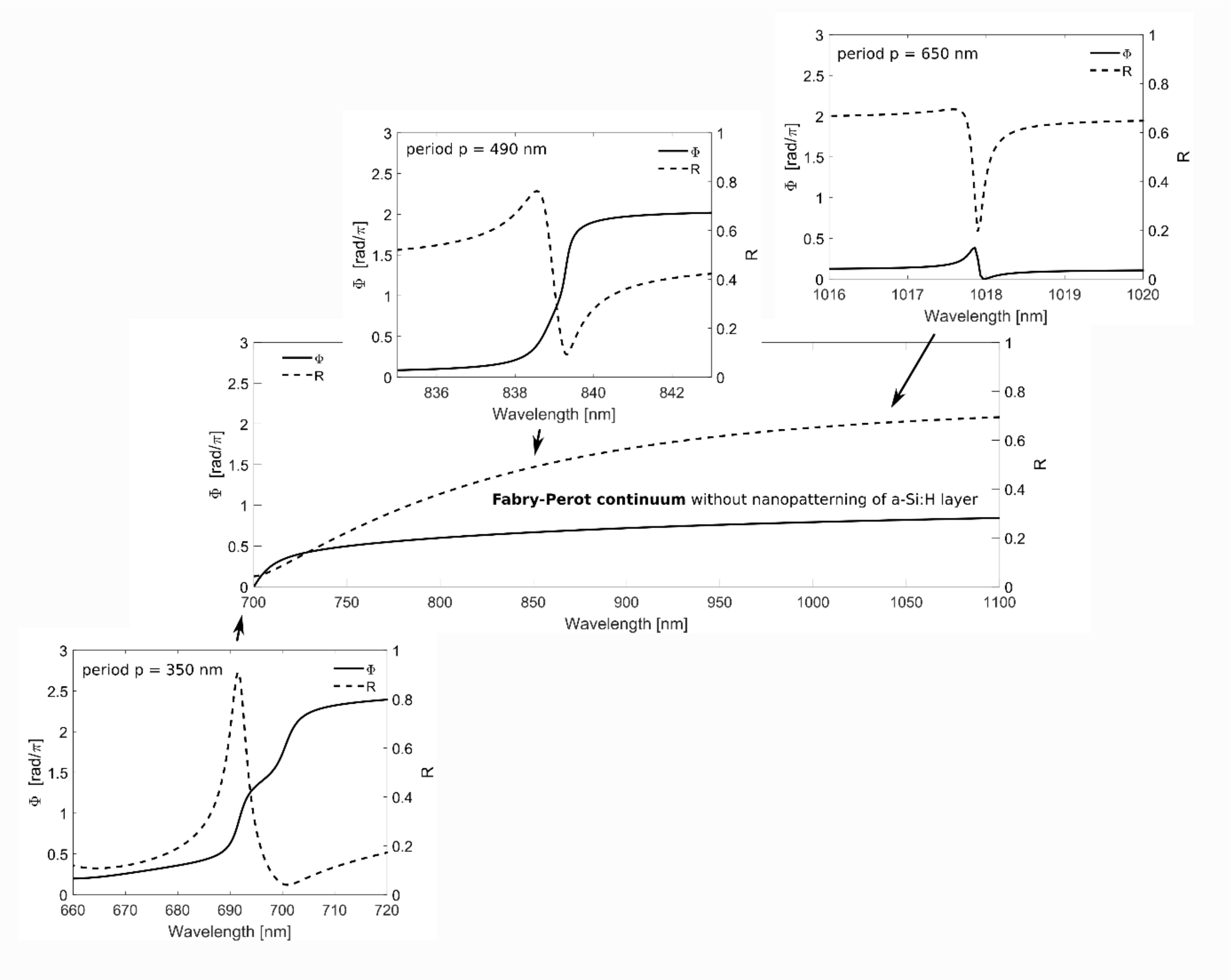


**Fig. S6**. **Simulated phase of various Fano resonances**. As a result of different overlap with the Fabry-Perot continuum. The middle inset at around 840 nm corresponds to the structure used in this work, see Fig. 2 in main manuscript.

### SI 3. Optical setup

The light source is a fiber coupled SLED (cBLMD-series Compact Broadband Light Source Modules, SUPERLUM) with a central wavelength of ~ 860 nm and a bandwidth of ~ 60 nm. The output is collimated and spatially coherent with a beam diameter of ~2mm. The bandpass filter with 1 nm FWHM was purchased from Andover Corp and is mounted on a rotatable base for wavelength tunability. The first (bottom) beam splitter is a polarizing beam splitter. The second (top) beam splitter directs 50% of the input towards the sensor chip and 50% of the resonantly reflected light towards the imaging lens. The axes of the nanohole array (x, y) are aligned at 45° with respect to the input polarization. The Wollaston prism (Thorlabs) splits orthogonally polarized light at an angle of 1°. The analyzer (linear polarizer) is placed in front of the compact CMOS camera (Thorlabs). The field-of-view is ~(2x2) $mm^2$.

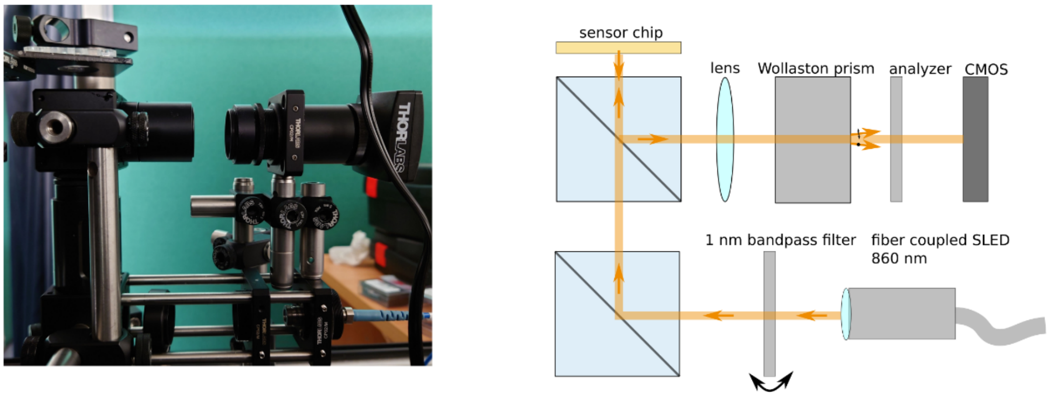


**Fig. S7**. **Interferometric optical setup.** Photograph and schematic of optical setup

## SI 4. Spectral bulk refractive index sensitivity

Using the Stanford Stratified Structure Solver ($S^4$)(51), the spectral shift in response to bulk refractive index changes is simulated and the results are fitted to determine an approximate bulk sensitivity of 150 nm/RIU for the TM mode.

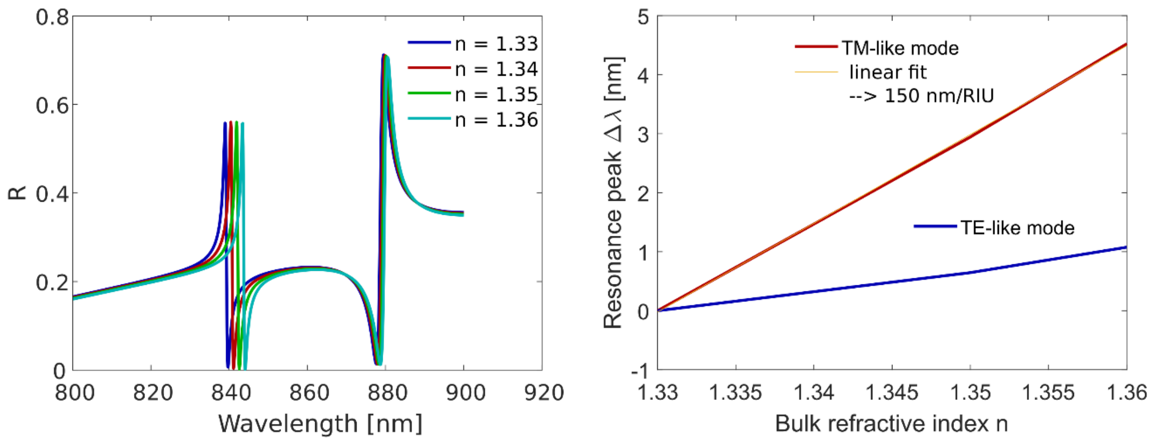


**Fig. S8**. **RCWA simulations of resonance wavelength shift** in response to bulk refractive index alterations of the upper cover medium (compare Fig. 2 in main manuscript) for the TM and the TE mode.

## SI 5. Langmuir isotherm fit of phase versus IP-10 concentration

Based on the Langmuir isotherm fit of the measured phase response to various IP-10 concentrations, the equilibrium constant $K_D$ is here approximately 60 nM.

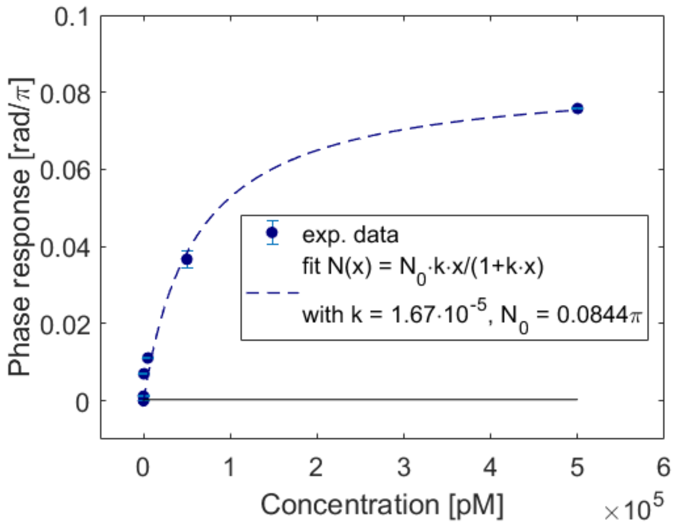


**Fig. S9**. **Isotherm fit.** Same data as shown in Fig. 4D in the main manuscript.

### SI 6. SLED spectrum

The SLED used for all the experiments shown in this work displays the spectrum below with a maximum intensity around 845 nm.

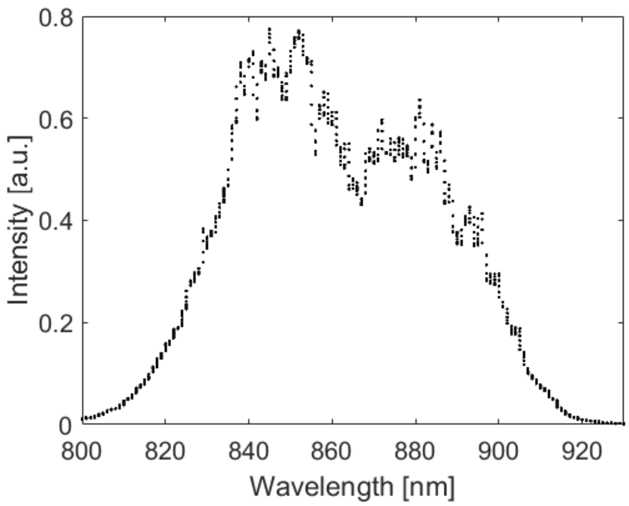


**Fig. S10**. **Spectrum of the SLED** used throughout this work

### SI 7. Phase response to bulk refractive index changes

To estimate the performance of any biosensor, the bulk refractive index sensitivity is typically investigated by introducing solutions of known refractive index and measuring the response, here the phase. Although the performance a sensor designed for protein sensing depends on the surface sensitivity rather than the bulk sensitivity, the bulk LOD is a first indicator of the expected biosensing performance. We here diluted glucose in $H_2O$ and measured the corresponding refractive index with a handheld commercial refractometer

(Reichert, Brix/RI-Chek) for calibration purposes. We took images of the corresponding interferogram over time for each concentration.

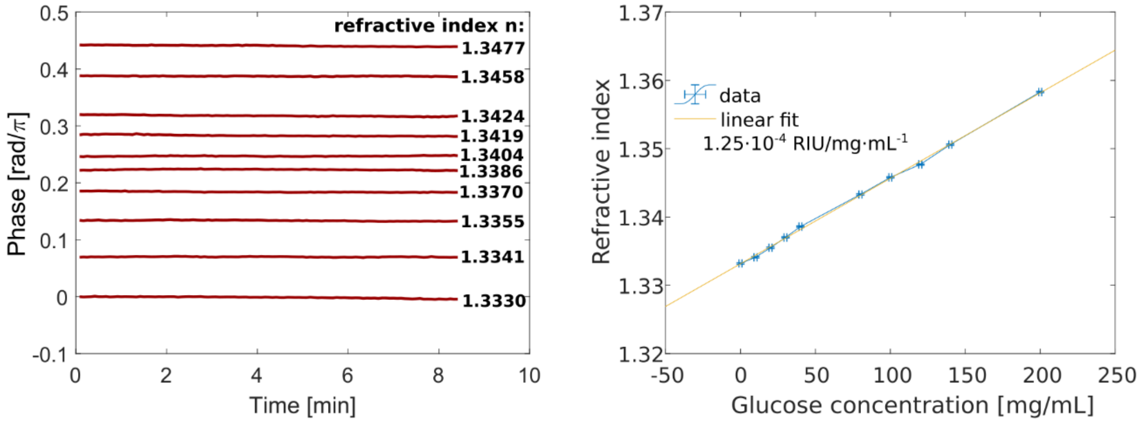


**Fig. S11**. **Bulk sensitivity.** To characterize the experimental bulk phase sensitivity, we exposed the sensor to glucose solutions of varying concentrations, which we calibrated using a commercial refractometer, and the corresponding phase responses are plotted together for ease of visualization. This is the data leading to the plot in Figure 3 B in the main manuscript.

## SI 8. Fano fit and Q-factor extraction

To extract the FWHM of the Fano peaks shown in Figure 2 in the main manuscript, we fit a Fano function:

$$F(\lambda) = a\,\frac{(q\cdot\sigma + (\lambda-\lambda_0))^2}{\sigma^2 + (\lambda-\lambda_0)^2} + e$$

Here, a is the amplitude, q the Fano parameter (*4*, *58*), $\sigma$ the peak half-width, $\lambda_0$ the peak wavelength and e an offset value. The FWHM is then approximated as FWHM = 2 $\sigma$ and Q = $\lambda_0$/ FWHM.

## SI 9. IgG sensing based on antibody spotting

Although the protein sensing results shown in the main manuscript are to be seen as proof-of-principle sensing to test the novel concepts of degenerate metasurface interferometry, we are mentioning the possibility of antibody spotting here, because spotting will allow highly multiplexed biomarker detection for clinical studies in the future.

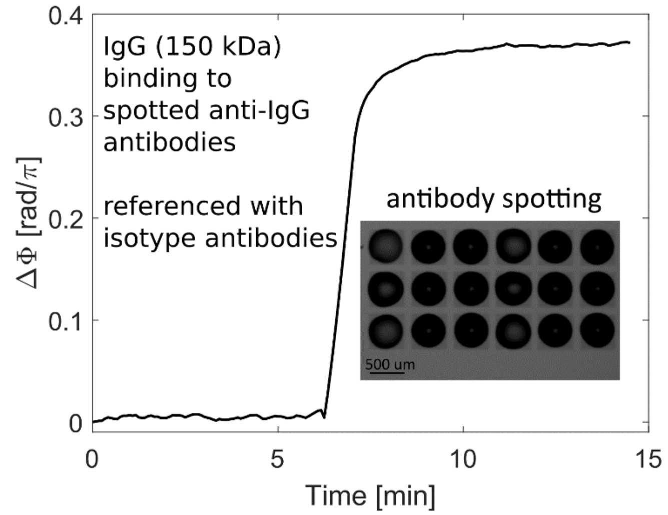


**Fig. S12**. **Antibody spotting.** Phase in response to flowing IgG over a surface (starting ~ 6 min after PBS baseline) functionalized with anti-IgG antibodies. Antibodies are spotted on the sensor surface using the sciFLEXARRAYERs S3 ultra-low volume dispensing system. The concentration of the antibody solutions in PBS was 500 ug/mL and both the specific anti-IgG antibodies and non-specific isotype antibodies were spotted next to each-other for the self-referenced interferometric read-out.

### SI 11. Interferogram contrast characterization

In the degenerate mode interferometry approach, the signal and reference beam have the same amplitude since they correspond to the same resonant mode, and the fringe visibility is therefore high with C ~ 80%. The resonant mode can also be interfered with the background rather than itself, which results in an unreferenced interferometry approach (mode – background, “sharp – flat”). The resulting contrast is lower (~ 40%) since the background intensity (reflection from the air-glass interface of the sample substrate and Fabry-Perot background from the a-Si:H slab) and the resonantly reflected light intensity are different. Note that the background signal (reflectance of sample area without nanopatterning) in an a-Si:H slab with its high refractive index of ~ 3.5 is higher than, e.g., a $Si_3N_4$ slab, which is useful since the background fringes can be used to monitor the existence of non-resonant drift in the same image as the resonant phase information.

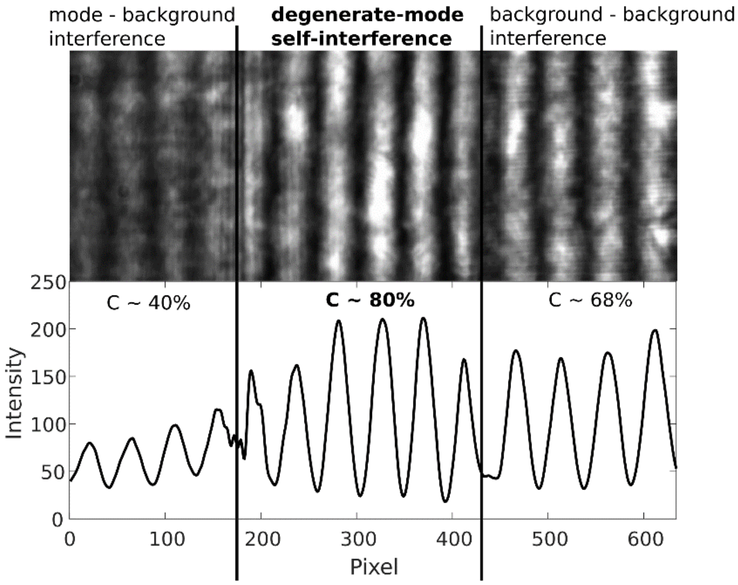


**Fig. S13**. **Interferogram contrast.** Image of 3 cases of interferograms on the same sample in the same FOV at 45° analyzer orientation. 1. Mode-background interference results in low contrast. 2. Self-interference results in high contrast because signal and reference beam are the orthogonal components of the same resonant mode and therefore have the same amplitude. 3. The background interferogram has an overall lower signal, but the contrast is also high since the orthogonal background components have approximately the same amplitude.

### SI 12. Table S1: Resonant interferometric sensing - literature review summary

The comparison presented in the table below is restricted to platforms with a similar degree of complexity and similar footprint as well as multiplexing capability and therefore limited to out-of-plane coupling platforms such as plasmonic nanohole arrays and dielectric photonic crystal slabs employing phase detection. We did not include waveguide-based platforms, e.g. (52), because there the superior overall LOD is based on the considerably higher phase sensitivity which is enabled by large-footprint waveguide arrangements without imaging capability.

| **Reference (Sorted by date) And platform** | **Light source** | **Noise σ** | **Sensitivity** | **LOD = 3σ/S [RIU]** | **Measured Proteins** |
|---|---|---|---|---|---|
| (53)<br><br>**Dielectric grating** | HeNe Laser + speckle reducer | "Noise" stated to be 10°<br>~ $1.8 \times 10^{-2}\,\pi$ | Not stated directly, here calculated (based on stated LOD) to be ~ 82 π/RIU | $6.8 \times 10^{-4}$ | X |
| (16)<br><br>**Gold nanohole array** | LED | OPD noise<br>3.9 nm | OPD sensitivity 9000 nm/RIU | $5.7 \times 10^{-4}$ | 500 ng/mL<br>-IgG 2<br>(150 kDa) |
| (20)<br><br>**Dielectric grating** | HeNe Laser | X | 6075 π/RIU * | X ** | X |
| (54)<br><br>**Same platform as (16)** | LED | OPD noise<br>3.9 nm | OPD sensitivity 9000 nm/RIU | $5.7 \times 10^{-4}$ | -CRP (~25 kDa)<br>18 ug/mL (estimated LOD)<br>-IL-6 (~25 kDa)<br>88 ug/mL (estimated LOD) |
| (*17*)<br><br>**Dielectric grating** | Laser diode | $1.8 \times 10^{-4}\,\pi$ | 289 π/RIU | $1.8 \times 10^{-6}$ | 1 pg/mL procalcitonin (13 kDa) |
| (55)<br><br>**Dielectric grating** | HeNe Laser | Detection limit stated to be 1°<br>→ $1.9 \times 10^{-3}\,\pi$ | X<br>Characterized in different work<br>5000°/RIU<br>~ 30 π/RIU | X<br>~$2 \times 10^{-4}$ | X |

| | | | | | |
|---|---|---|---|---|---|
| (*18*)<br><br>**Dielectric grating** | HeNe laser | 4.75 x $10^{-3}$ rad =<br>1.5 x $10^{-3}$ π | 420rad/RIU = 134 π/RIU | $3.4 \times 10^{-5}$ | 76 nM (2.3ug/ml) $\alpha$-thrombin (37 kDa) |
| (56)<br><br>**Dielectric grating** | Laser + lock-in amplifier | X | $3.73 \times 10^{4}$ °/RIU<br><br>= 207 π/RIU | X *** | X |
| (57)<br><br>**Dielectric grating** | HeNe laser | 3 x $10^{-3}$ rad = 9.5 x $10^{-4}$ π | 800 rad/RIU= 255 π/RIU | Not given, here calculated: $1.1 \times 10^{-5}$ | $\alpha$-thrombin<br><br>streptavidin concentration of 8 μg/ml |
| **This work**<br><br>**Dielectric nanohole array** | LED | 9.2 x $10^{-5}$ π | 230 π/RIU | $1.2 \times 10^{-6}$ | 50 pM<br>IP-10/ CXCL10 (9 kDa) |

* We were unable to replicate this value of phase sensitivity when simulating the author's structure
** $3.43 \times 10^{-7}$ was obtained with system resolution, not 3σ noise, and is therefore not the relevant LOD based on 3σ.
*** authors quoted statistical error phase of lock-in amplifier 0.01◦, → $2.68 \times 10^{-7}$ RIU. This is not the LOD based on 3σ.

### SI 13. Noise reduction due to self-referencing in degenerate interferometry case

The schematic below illustrates how self-referencing based on mode degeneracy (phase noise matching), the referencing of equal resonances, results in lower noise compared to typical system of two resonances that are each referenced with a broad background and then referenced with each other, where the noise adds up due to signal subtraction.

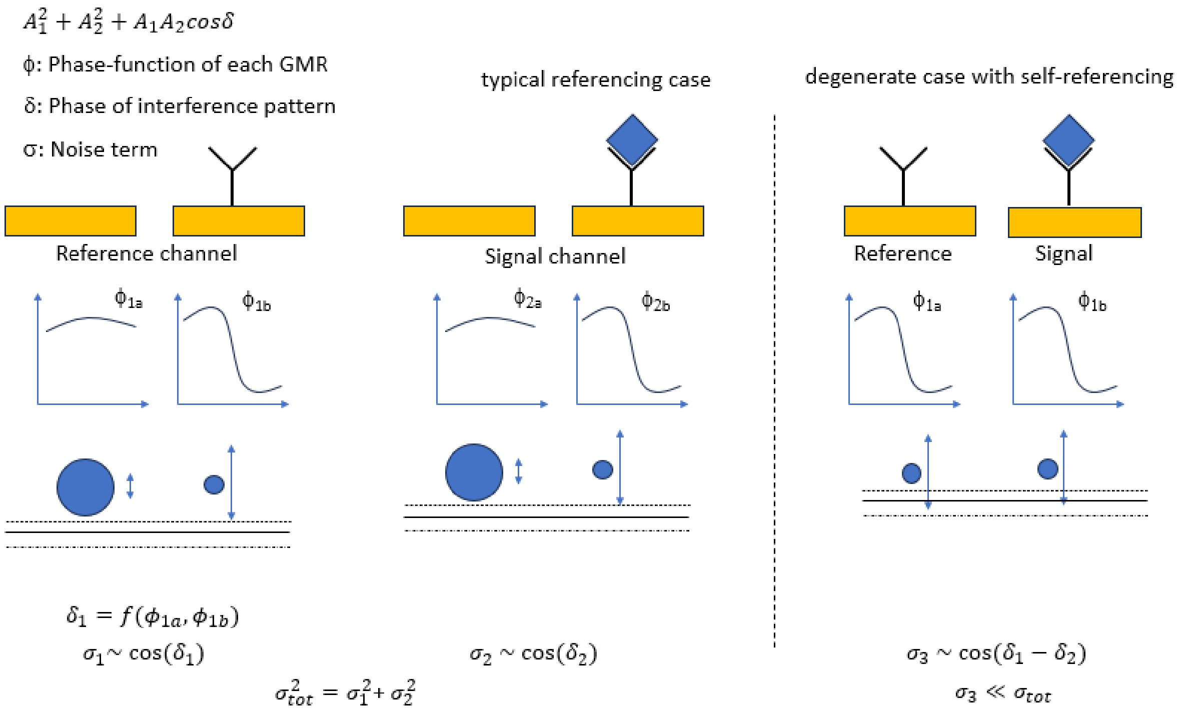


**Fig. S14**. **Resonant phase noise matching.** Schematic explanation of noise reduction due to self-referencing of two equal resonance components versus a typical referencing case of a resonance with a flat background or broad resonance.

### SI 14. Material index effect on surface sensitivity

The simulations are based on the assumption of a protein refractive index of 1.45 and homogeneous, dense protein layer. The goal is not to quantify expected resonance shifts upon protein binding, but rather to illustrate the effect of the material index on the surface sensitivity to explain why a-Si:H was chosen for this work.

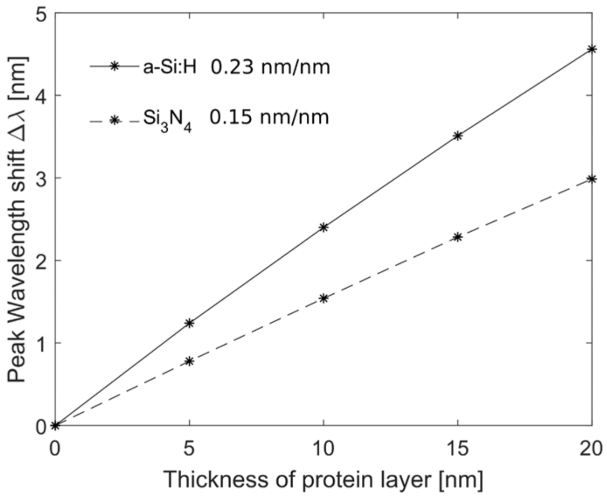

**Fig. S15. Surface sensitivity.** Simulation of resonance shift in response to increasing the thickness of a 'protein layer', approximated as homogeneous layer with refractive index of 1.45. The TM modes supported by a a-Si:H and a Si3N4 nanohole array at the same wavelength are compared in terms of their surface sensitivities.

### SI 15. Images of chirped nanohole array for degeneracy-maintaining alignment

Figure S16 shows an image of a 'normal' squared lattice nanohole array at resonance (bright square on the top left) and a 'chirped' nanohole array for alignment on the same chip (bottom right), here in the same FOV. The chirped nanohole array can be used to ensure the degeneracy, since $k_x \neq k_y$ results in a 'splitting' of the resonance line. See our work on chirped nanohole arrays (6) for more information. The period is tuned in space leading to a tuned resonance condition, which is now fulfilled where the resonance is observed as bright lines. A lifting of the mode degeneracy results in 'splitting' of this resonance line as seen here. Therefore, the chirped approach can be used to ensure an alignment leading to an equal excitation of the degenerate modes at the Γ-point for degenerate mode interferometry.

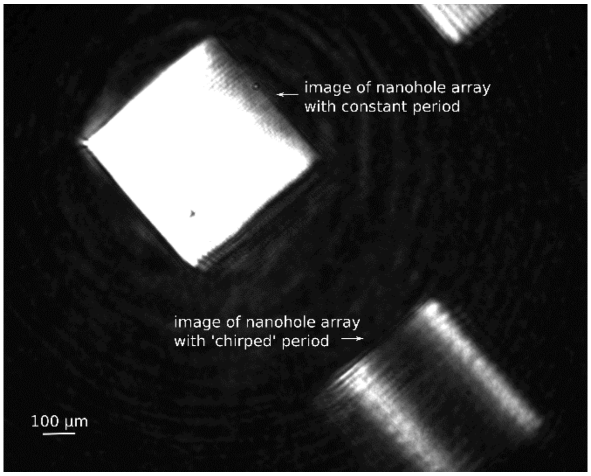


**Fig. S16. 'Chirped' nanohole array for alignment.** Image of nanohole array at resonance with constant period leading to same reflectance over the whole sensor area (top left). This configuration is used for the interferometry work. In the same FOV (bottom right) a chirped nanohole array is used for alignment purposes to ensure mode degeneracy for interferometry. Note that the intensity scale is adjusted to show the chirped nanohole array clearly, leading to an overexposure of the top left resonance.

### SI 16. Noise analysis – "pink" camera noise indication

The self-referencing based on degenerate mode interferometry reduces the system-noise to a low level of $\sigma = 9.2 \cdot 10^{-5}\,\pi$. To characterize the nature of this remaining noise, we perform a Fourier transform of the noise signal and compare this signal to the Fourier transform of both white noise and pink noise, where pink noise is the white noise divided by the frequency f. The white noise shows a flat response, as expected. Plotted on a loglog scale, it is evident from Fig. S17 C, that the remaining resonant noise ("sharp – sharp") does not show a white noise behavior, but rather a frequency dependent behavior, similar to the pink noise. This result is an indication that the remaining noise might limited by the CMOS itself (*44*, *45*).

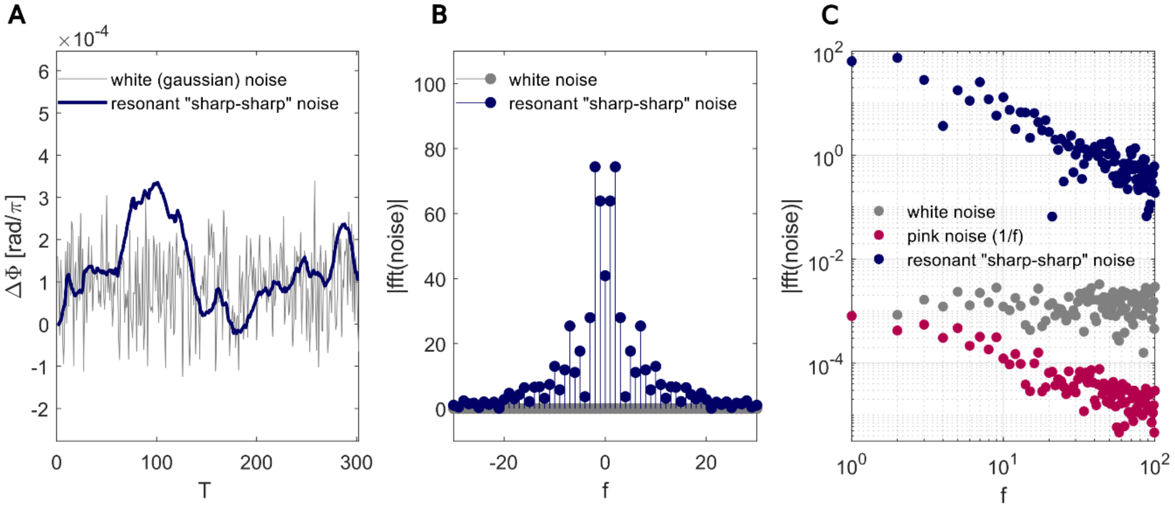


**Fig. S17. Noise characterization.** White noise with gaussian profile was generated (MATLAB randn function) to compare this to the remaining noise of the self-referenced phase information (Fig. 3 main manuscript). The Fourier transform of the white noise displays a flat response, while pink noise (white noise multiplied with 1/f) shows a linear frequency dependence when plotted on a loglog scale. The resonant "sharp-sharp" noise shows a similar trend.

## SI 17. Movie S1 - caption

Movie S1 displays how the interferogram fringes respond to wavelength tuning of the incident light as the resonance goes through an almost complete cycle. On the left, the video is showing the spectrum of the excitation beam measured with a spectrometer. The tuning of the peak wavelength is achieved via rotation of the bandpass filter (SI 3) and the well-known Fabry-Perot resonance dependence on the angle of incidence. On the right, the video shows the corresponding interferogram for each of these incidence peak wavelengths recorded with the CMOS camera. Specifically, the main, centered part of the displayed image represents a nanohole array going through the resonance cycle, while the top and bottom parts of the image show the background (un-patterned silicon surface). Since the phase noise matching approach that we employ for specific protein sensing would result in zero detectable phase shifts when tuning the incident wavelength (both mode components depend equally on the wavelength), we here sheared the reflected beams such that the resonant nanohole array overlaps with the non-resonant background to extract the phase-wavelength dependence. It is evident that the background fringes drift in the opposite direction with respect to the resonant phase of the nanohole array. We therefore reference the resonant phase wavelength dependence with these background fringes to extract the phase behavior (i.e., Fig. 3 A). Note that we did not adjust the analyzer orientation throughout the wavelength tuning, meaning that the contrast of the interferogram reduces while going through the peak of the resonance in this case, since the reflectance increases while the background remains constant. When using the phase noise matching approach, the contrast is optimal since both orthogonal components have similar reflectance values even throughout a protein sensing experiment, since very small resonance changes are detected that do not result in large changes in relative resonance amplitudes of the two components. Further note that we have rescaled all images contained in the video separately such that the absolute intensities are her not representative of the resonance amplitude behavior.
